# Incorporating Biological Pathways via a Markov Random Field Model in Genome-Wide Association Studies

**DOI:** 10.1371/journal.pgen.1001353

**Published:** 2011-04-07

**Authors:** Min Chen, Judy Cho, Hongyu Zhao

**Affiliations:** 1 Division of Biostatistics, Department of Clinical Sciences, University of Texas Southwestern Medical Center, Dallas, Texas, United States of America; 2 Internal Medicine, Yale University, New Haven, Connecticut, United States of America; 3 Center for Statistical Genomics and Proteomics, Department of Epidemiology and Public Health, Yale University, New Haven, Connecticut, United States of America; University of Alabama at Birmingham, United States of America

## Abstract

Genome-wide association studies (GWAS) examine a large number of markers across the genome to identify associations between genetic variants and disease. Most published studies examine only single markers, which may be less informative than considering multiple markers and multiple genes jointly because genes may interact with each other to affect disease risk. Much knowledge has been accumulated in the literature on biological pathways and interactions. It is conceivable that appropriate incorporation of such prior knowledge may improve the likelihood of making genuine discoveries. Although a number of methods have been developed recently to prioritize genes using prior biological knowledge, such as pathways, most methods treat genes in a specific pathway as an exchangeable set without considering the topological structure of a pathway. However, how genes are related with each other in a pathway may be very informative to identify association signals. To make use of the connectivity information among genes in a pathway in GWAS analysis, we propose a Markov Random Field (MRF) model to incorporate pathway topology for association analysis. We show that the conditional distribution of our MRF model takes on a simple logistic regression form, and we propose an iterated conditional modes algorithm as well as a decision theoretic approach for statistical inference of each gene's association with disease. Simulation studies show that our proposed framework is more effective to identify genes associated with disease than a single gene–based method. We also illustrate the usefulness of our approach through its applications to a real data example.

## Author Summary

Statistical methods used in most GWAS are based on the analysis of single markers. Prior biological information about markers, genes, and pathways is not commonly incorporated in the detection of associated disease loci. Recently a number of methods have been developed to incorporate such information, and it has been shown that they may make use of prior biological knowledge in association analysis. However, most of these methods ignore the regulatory relationships and functional interactions among genes. In this article, we propose a statistical method that can explicitly model the interactions of genes in a neighborhood defined by the topology of a pathway. Simulation studies and a real data example show that the proposed method can improve the power of identifying associated genes when they are in the neighborhood of other genes whose association has been firmly established in previous studies.

## Introduction

In genome-wide association studies (GWAS) researchers examine a large number of markers across the genome in many individuals to identify associations between genetic variants and disease, or to prioritize markers for follow up studies. However, most of the times the signals from individual markers are weak and the sample size is not large enough to have adequate power for true discoveries, especially when the minor allele frequency is low. Various approaches have been developed to increase statistical power, including aggregating multiple markers from the same gene or in the same haplotype block region and incorporating information from other sources into the GWAS analysis. It has been found that the gene level analysis has the ability to identify new associations in addition to those identified using individual Single Nucleotide Polymorphisms (SNPs) [1], [2]. Gene-based analyses include those using the most significant SNP within and near a gene [1]; combination statistics (Fisher, Sidat, and Simes) from all individual markers [2]; Principal Component Analysis (PCA) regressions [3] and the sparse partial least squares regressions [4]. To incorporate prior biological knowledge, one information rich resource is biological pathways. It is believed that genes interact with each other in biological processes, and it is conceivable that they may jointly affect the risk of a complex disease. There exist an abundance of databases containing known gene pathways and protein-protein interactions, such as KEGG, BioCarta, GenMAPP, and HPRD. A number of gene prioritization methods incorporating prior biological knowledge have been developed for GWAS. Some examples include Prioritizer [5], Endeavour [6], CGI [7], CANDID [8], GeneWanderer [9], CIPHER [10], GIN [11], and the pathway based gene set enrichment approach [1]. These methods have shown that incorporating prior biological information in GWAS is useful. However, they do not consider functional relationships among genes. The general input of these approaches is a list of genes as a set, in which genes are treated as exchangeable without taking into account the regulatory relationships among them. As a result, information from the pathway topology and interactions among genes is usually ignored. However, how genes are functionally related to each other in a pathway may be very informative for GWAS analysis and such information can be utilized to increase the power of detecting real associations. When associations have been firmly established for some genes either through GWAS or prior candidate gene-based studies, we can take advantage of this knowledge to examine other genes related to these known genes through the same pathways they all participate in.

In this paper we propose a Markov Random Field (MRF) model to incorporate biological pathway information in GWAS. MRF has been considered by several authors to combine data from different sources in genomics studies, e.g., a spatial normal mixture model [12] for gene expression and CHIP-chip data, a Gamma-Gamma model and MRF for mRNA microarray data [13], and prioritizing genes by combining gene expression and protein interaction data [7]. However, little has been done in the context for GWAS, with the exception of Li et al. [14] who proposed a hidden MRF for GWAS. But their method is developed in the context of jointly analyzing markers in linkage disequilibrium.

**Figure 1:**
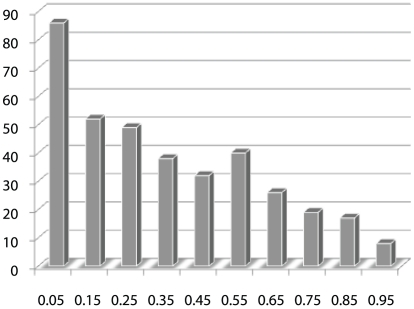
Histogram of *p* values of 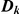, the number of edges connecting a pair of “interesting” genes in a pathway 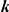, which depends on the labels of all genes. A large value of 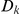 would suggest that “interesting” genes are more likely to be neighbors. A permutation procedure is used to derive an empirical distribution of 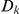 under the null hypothesis. The 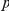 value of an observed 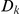 is calculated with respect to this empirical distribution. See the Introduction section for more details.

We first present a motivating example from a GWAS of Crohn's disease [15] for the proposed method. As will be shown next, the result clearly suggests that genes in the same neighborhood within a pathway tend to show similar association status. This Crohn's disease cohort includes 401 cases and 433 controls, and the Illumina HumanHap300 BeadChip (Illumina, San Diego) were used for genotyping. We first mapped SNPs to genes and then applied PCA regressions to obtain gene-level *p* values of the association tests with Crohn's disease status [3]. More details about this data set are given in the Materials and Methods section. We then obtained pathway and interaction from BioCarta (http://www.biocarta.com/), GeneMAPP [16] and KEGG [17]. We consider a total of 3,735 genes in over 350 pathways. Genes on the same chromosome that are within 1 million base pairs are excluded to avoid effects caused by possible linkage disequilibrium. To see whether genes connected with each other in the same pathway tend to show similar evidence for association, we use a cut-off value 0.15 where genes whose *p* values are below this cut-off are considered interesting and labeled with 1. Note that we use a relatively loose threshold so that a sufficiently large number of genes are called “interesting” and this loose cut-off also reflects our belief that many genes have weak effects and only show moderate evidence of association. In a pathway 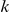, we consider the number of edges connecting a pair of “interesting” genes, which depends on the labels of all genes. We denote this number by 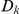. A large value of 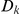 would suggest that “interesting” genes are more likely to be neighboring genes. To assess the statistical evidence for the tendency to observe large 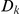 values, we employ a permutation procedure as follows. In each permutation, we randomly permute the “interesting” labels of all genes and derive a permuted statistic and these permuted statistics are used to arrive at an empirical distribution of 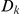 under the null hypothesis that there is no tendency for neighboring genes to have similar disease association status, i.e. “interesting” or not. We then compare the observed 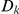 statistic with the empirical distribution. Finally the *p* value of the observed 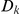 in this empirical distribution is calculated. A *p* value close to 0 indicates that “interesting” genes tend to be neighbors. This procedure is repeated for all pathways, and the histogram of *p* values of 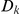 for all pathways is plotted in Figure 1. It is evident that this distribution is highly skewed to the left, which suggests associated genes tend to be neighbors in a given pathway.

In the rest of this article, we first introduce our model and statistical inferential procedures. The performance of our methods is then assessed through both simulation studies and real data applications.

## Results

### A MRF model of gene pathways

We start by considering a simple model in which a pathway is represented by an undirected graph 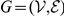 where 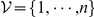 is a set of 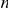 genes (nodes) and 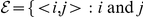 are directly connected} denotes the set of all edges. For the 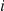
th gene in 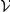, let 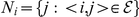 denote the set of its neighbors, and 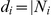 denote the number of its neighbors. Let 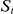 denote the true association status where 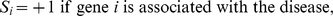


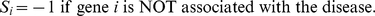



The values 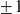 are referred to as labels of a node hereafter. Let 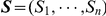 denote the labeling of 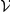. Thus 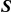 is a spatial random vector whose elements may be correlated with each other. Note that each node can be labeled either 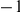 or 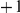, and so there are a total of 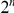 unique configurations of the pathway. The ultimate goal is to infer the value of 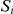 based on the pathway topology and the observed association data.

To formalize the idea that neighboring genes tend to have similar association status, we need a probability measure so that nodes connected with each other tend to have the same labels. Here we consider a nearest neighbor Gibbs measure [18] that has the following form:
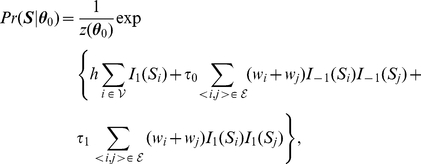
(1)where 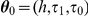 are the prior parameters or hyperparameters, 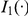 and 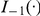 are the indicator functions, 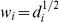, and 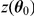 is a normalizing function that is the sum over all 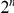 possible configurations:
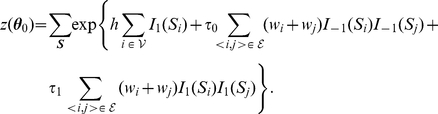
(2)


Note that it is prohibitive to evaluate 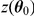 when 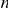 is large. Here 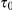 and 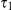 assign prior weights to edges connecting two non-associated nodes and two associated nodes, respectively. The function 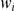 will be elaborated in more details in the context of the conditional probability later.

In (1), the second sum is taken over all edges connecting direct neighbors in which both end nodes are labeled −1, and the third sum is taken over all edges in which both end nodes are labeled 1. Positive 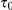 and 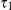 will put more weights on configurations in which directly linked nodes have the same labels, which is desirable in our context. The hyperparameter 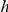 determines the marginal probability of 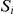 when 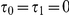, i.e., all nodes are treated as singletons that are independent: 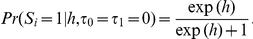



The simple form Gibbs measure in (1) has the Markov property that makes it attractive to model a biological pathway, in which directly linked genes interact with each other. It defines a MRF, which by definition is a probability measure that satisfies 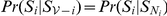, where 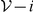 denote all nodes but 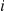, and 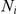 is the set of all direct neighbors of node 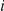. Please see Materials and Methods for details.

### Posterior distribution

Now we discuss the posterior distribution of association status after combining the evidence from the observed association statistics at the gene level and the structure of the gene pathway. Before we proceed, it is necessary to present the likelihood function of the observed data. We consider the situation where the observed evidence of association is summarized by 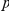 values, which are assumed to be conditionally independent given the true association status 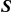. Under the null hypothesis of no association, each *p*-value has a uniform (0,1) distribution. In this article, we consider 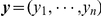, where 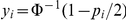 and 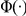 is the CDF (Cumulative Distribution Function) of 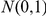. Therefore, under the null hypothesis of no association, i.e., 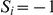, the density of 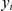 is 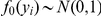. However, if there is association between the gene and disease, i.e., 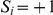, the distribution of 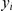 is usually unknown. For simplicity, we assume that it is from 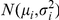, where 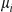 is the location parameter and 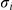 is the scale parameter that usually depends on the true effect size, allele frequencies, and the sample size. To account for the uncertainty about the parameters, we can put prior distributions on 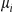 and 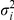, and marginalize over them to obtain the predictive density of 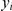. Here we consider conjugate priors 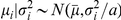 and 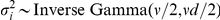, or 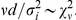 We denote 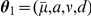 that are hyperparameters. The prior mean of 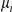 is 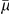 and its variance is 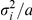. The prior mean of 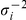 is 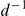 and the prior variance is 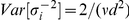. This prior is of conjugate form so that the integration over 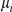 and 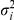 is analytically tractable. We note that the hyperparameters can be estimated from the observed data via an empirical Bayes method (see Text S2, Figures S1 and S2). Under this prior setting, the marginal density of 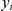 is 
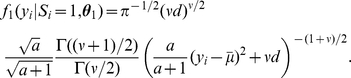



This is equivalent to 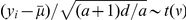 when 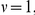 2, and others.

The joint marginal density of 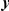 is
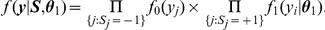



Thus, the posterior distribution of 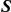 given the observed data 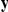 is 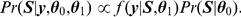
(3)


Similar to the MRF interpretation of the prior distribution (1), the posterior also has a nice conditional distribution and is actually a MRF, as will be shown in the Materials and Methods section.

When 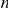 is large, since it is prohibitive to evaluate posterior probabilities on the entire space of configurations, we implement a Markov chain Monte Carlo (MCMC) method to sample from the posterior distribution. Naturally a Gibbs sampler is well suited for a MRF. As will be shown later, due to the MRF property, the posterior has a nice closed-form conditional distribution that can greatly facilitate the MCMC.

### Making inference based on the posterior distribution

Most GWAS lead to a set of candidate genes/SNPs that will need to be validated in follow-up studies. Therefore, it is important to include as many truly associated genes as possible among the top ranked genes. Our proposed method allows us to rank order genes as detailed below.

There are several ways of inferring the labels according to the posterior distribution of 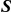. The first one is to use maximum *a posteriori* (MAP) estimate, which is the configuration with the largest posterior probability, a reasonable point estimate for 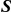. Let us denote it by 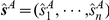. The MAP is the maximizer of the joint posterior distribution:
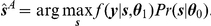



A Gibbs sampler outlined above can be applied to stochastically search for the solution to the above optimization problem. Multiple restarts with different initial configurations are recommended. An alternative approach is to base the estimate on the posterior conditional probability of 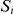 given the observed data and all the other nodes 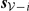. We can estimate 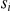 by maximizing this conditional probability (MCP): 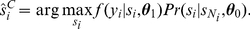
(4)


The advantage of this approach is that the above problem is trivial to solve. As will be explained in equation (8) of the Materials and Methods section, the second term in formula (4) can be evaluated in closed form. Besag [19] proposed an algorithm known as iterated conditional modes (ICM) that iteratively updates 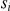. Note that the convergence of ICM is assured because the posterior is proportional to 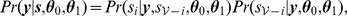
which never decreases at any iteration because the first term is non-decreasing and the second one is a constant. So it is easy to see the ICM will converge to a local maximum in the posterior distribution. Since the ICM runs fast and usually converges in several iterations, multiple restarts with different initial configurations are recommended. Finally the resulting configurations can be compared by evaluating 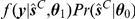 up to a normalizing constant to pick the largest one.

The inference can also be based on the marginal posterior probability. Let 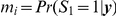. We consider a decision rule in the form 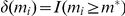, where 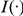 is an indicator function and 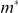 is the sought decision threshold. If 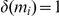, the decision is positive (also referred to as discovery) and gene 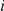 is called to be associated with the disease. Likewise if 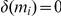 the decision is negative. To address the problem of multiple comparisons, we consider loss functions associated with making wrong decisions (false discoveries and false negatives), and solve the decision problem by minimizing the expectation of the loss functions under the posterior distribution. Here we consider two loss functions. First, if we are interested in the 0-1 loss function 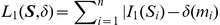, we may want to minimize the expected loss 
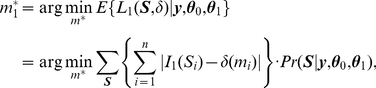
(5)under the posterior distribution of 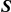. The solution is 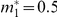. Note that 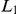 assigns equal loss to the false positive and false negative errors. This is to minimize the expected frequency of making wrong calls for the association status. Note that the performance of the decision rule 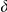 is based on the frequentist operating characteristic in the Bayesian framework, which is common in medical decision makings [20]. The second loss function we consider is the false discovery rate (FDR): 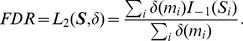
(6)


Suppose the goal is to control the expected FDR, under the posterior distribution, such that it is no more than 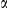, i.e., 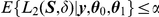. If we rank order all genes by their posterior probabilities from the largest to the smallest, and let 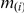 denote the 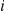
th order statistics, then the solution is to choose a cut off value 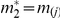 where 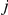 is the largest integer that makes 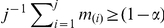. We should mention that more complicated loss functions can be considered under the framework of our model. See Müller et al. [20] for other examples.

### Simulation studies

**Figure 2:**
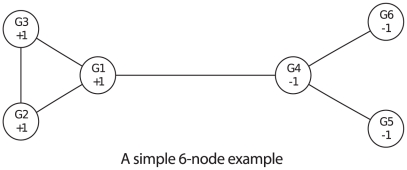
A simple 6-node network.

First we use simulated data to study the performance of the proposed method. The simulation is based on a simple 6-node network shown in Figure 2. Genes G1 through G3 are assumed to be associated with the disease (labeled +1) while G4 through G6 are not associated with the disease (labeled −1). Data are simulated from a disease model as follows. We assume G1, G2 and G3 have independent effects on disease risk and each has a disease related SNP. The genotypes and minor allele frequencies of these three SNPs are denoted by 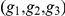 and 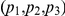, respectively, where 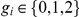 for 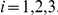 A multiplicative genetic model is assumed for the risk of having the disease. More specifically, for an individual with genotype 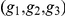, the risk is 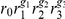, where 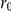 is the baseline risk of those carrying two normal alleles in all three genes, and 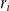 is the relative risk, or effect size, of gene 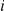, 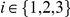. For each SNP the Hardy-Weinberg equilibrium (HWE) is assumed to hold in the general population so that the genotype probabilities are 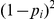, 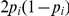, and 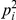 for 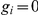, 1, and 2, respectively. In the simulation we use three minor allele frequencies 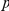 = (0.05, 0.10, 0.15), three disease prevalence values 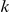 = (0.05, 0.10, 0.15), and six effect sizes 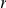 = (1.05, 1.10, 1.15, 1.20, 1.25, 1.30). As a result, there are a total of 54 settings of 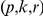, for each of which we first let 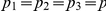 and 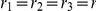, and then calculate the baseline risk 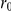, and finally obtain the conditional distribution of the genotypes 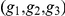 given the disease status. Then genotypes of G1, G2 and G3 of 600 cases and 600 controls are simulated according to the conditional genotype distribution. The 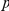 values of the three causal genes are calculated from a logistic regression of the data. For G4 through G6, the 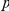 values are simulated from Uniform(0,1). The power of detecting the true association depends on the disease model. In this case, larger values of relative risk, MAF and prevalence corresponds to association tests with higher power.

In the simulation we set the hyperparameters 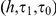 = (−1, 0.25, 0.01) where more weights are assigned to edges connecting two associated genes. This corresponds to a prior belief that the probability of association is roughly between 0.35 and 0.50. The hyperparameters 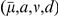 are set to (3, 1, 10, 1) where a large value of 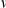 puts a large prior variance on 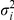, which allows a wide range of values for both 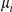 and 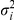. For each simulated data set, the posterior probabilities are enumerated since there are only 64 possible configurations in this simple example. The simulation is repeated 500 times. We compare the proposed method using the posterior mean with the one using the 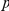 value, and apply cut-off values of 0.7 and 0.05 for posterior probabilities and 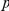 values, respectively. For each simulated data set, we calculate the false positive rate (FPR), sensitivity (Sens.), and false discovery rate (FDR) by thresholding on 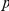 values and posterior probabilities. In addition, genes can be rank ordered by the two methods and the area under the Receiver Operating Characteristic curve (AUC) can be calculated. The average values of the three rates plus the AUC over the 500 simulated data sets are shown in Table 1. As can be seen, the proposed method of the posterior probability has higher sensitivity, smaller false discovery rate, and higher AUC than the 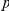 value thresholding in every setting of the prevalence, MAF and effect size, while the FPR of both methods are controlled at 0.05.

**Table 1:** Average FPR, sensitivities, FDR, and AUC of the 6-node network.

Prevelance	Effect Size		MAF = 0.05				MAF = 0.10				MAF = 0.15			
		Method	FPR	Sens.	FDR	AUC	FPR	Sens.	FDR	AUC	FPR	Sens.	FDR	AUC
0.05	1.05	*p* value	0.05	0.06	0.42	0.50	0.05	0.07	0.39	0.52	0.04	0.08	0.35	0.54
		Posterior	0.04	0.07	0.39	0.55	0.04	0.08	0.35	0.56	0.04	0.08	0.33	0.59
	1.10	*p* value	0.05	0.08	0.38	0.55	0.05	0.12	0.25	0.60	0.05	0.13	0.26	0.60
		Posterior	0.05	0.08	0.38	0.60	0.04	0.13	0.22	0.65	0.04	0.16	0.21	0.67
	1.15	*p* value	0.05	0.13	0.25	0.60	0.04	0.20	0.18	0.67	0.06	0.28	0.17	0.72
		Posterior	0.04	0.15	0.21	0.67	0.04	0.23	0.16	0.74	0.05	0.33	0.13	0.80
	1.20	*p* value	0.05	0.18	0.19	0.64	0.04	0.32	0.10	0.76	0.05	0.40	0.11	0.81
		Posterior	0.05	0.20	0.17	0.71	0.04	0.38	0.09	0.84	0.05	0.49	0.09	0.88
	1.25	*p* value	0.05	0.25	0.16	0.70	0.06	0.41	0.12	0.80	0.05	0.56	0.07	0.88
		Posterior	0.05	0.29	0.14	0.77	0.06	0.51	0.10	0.87	0.06	0.68	0.06	0.93
	1.30	*p* value	0.05	0.34	0.11	0.76	0.05	0.57	0.07	0.88	0.05	0.72	0.05	0.92
		Posterior	0.05	0.41	0.10	0.83	0.06	0.68	0.06	0.93	0.05	0.83	0.05	0.96
0.1	1.05	*p* value	0.05	0.06	0.43	0.54	0.05	0.08	0.39	0.52	0.05	0.07	0.41	0.53
		Posterior	0.05	0.07	0.42	0.58	0.05	0.08	0.39	0.58	0.05	0.07	0.38	0.59
	1.10	*p* value	0.05	0.09	0.34	0.57	0.04	0.13	0.24	0.60	0.05	0.16	0.25	0.62
		Posterior	0.04	0.09	0.31	0.62	0.04	0.15	0.20	0.66	0.05	0.18	0.21	0.70
	1.15	*p* value	0.04	0.14	0.22	0.62	0.05	0.21	0.18	0.67	0.05	0.27	0.14	0.72
		Posterior	0.04	0.15	0.20	0.69	0.05	0.24	0.16	0.73	0.05	0.33	0.11	0.80
	1.20	*p* value	0.04	0.21	0.16	0.68	0.05	0.33	0.12	0.77	0.05	0.45	0.09	0.82
		Posterior	0.04	0.24	0.14	0.76	0.06	0.40	0.11	0.84	0.06	0.54	0.08	0.89
	1.25	*p* value	0.05	0.27	0.13	0.73	0.05	0.48	0.08	0.84	0.04	0.62	0.05	0.91
		Posterior	0.04	0.33	0.11	0.80	0.05	0.57	0.06	0.90	0.05	0.73	0.05	0.95
	1.30	*p* value	0.05	0.37	0.12	0.79	0.05	0.61	0.05	0.90	0.05	0.78	0.04	0.94
		Posterior	0.05	0.46	0.09	0.87	0.05	0.73	0.05	0.94	0.05	0.87	0.04	0.97
0.2	1.05	*p* value	0.06	0.06	0.50	0.52	0.05	0.08	0.39	0.54	0.06	0.09	0.39	0.55
		Posterior	0.05	0.06	0.49	0.55	0.04	0.08	0.33	0.59	0.05	0.09	0.36	0.59
	1.10	*p* value	0.06	0.09	0.39	0.53	0.05	0.13	0.26	0.60	0.06	0.17	0.24	0.64
		Posterior	0.05	0.09	0.36	0.58	0.04	0.15	0.22	0.66	0.04	0.20	0.18	0.71
	1.15	*p* value	0.05	0.15	0.24	0.63	0.04	0.24	0.14	0.71	0.04	0.37	0.10	0.78
		Posterior	0.05	0.18	0.20	0.69	0.05	0.28	0.13	0.79	0.05	0.44	0.10	0.85
	1.20	*p* value	0.05	0.24	0.18	0.70	0.05	0.43	0.10	0.81	0.04	0.55	0.06	0.89
		Posterior	0.05	0.28	0.15	0.78	0.05	0.52	0.08	0.88	0.05	0.67	0.06	0.93
	1.25	*p* value	0.05	0.35	0.12	0.78	0.05	0.57	0.06	0.88	0.04	0.73	0.04	0.94
		Posterior	0.05	0.43	0.09	0.84	0.05	0.68	0.05	0.94	0.05	0.82	0.04	0.97
	1.30	*p* value	0.05	0.46	0.08	0.83	0.05	0.73	0.05	0.93	0.05	0.86	0.04	0.97
		Posterior	0.06	0.57	0.08	0.90	0.06	0.84	0.05	0.97	0.05	0.93	0.04	0.99

**Figure 3:**
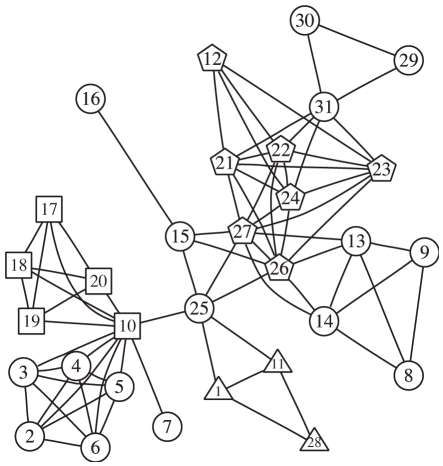
A 31-node network adapted from BioCarta “Human Rho cell motility signaling pathway.” Triangles, rectangles and pentagons denote three different sets of truly associated genes, each of which contains three, five, and seven nodes, respectively.

The second simulation study is based on the network shown in Figure 3. This network was adapted from BioCarta “Human Rho cell motility signaling pathway” and we deleted a few genes that are either absent from our Crohn's disease data or not connected to others. We assume three different sets of truly associated genes, plotted in triangles, rectangles and pentagons, each of which contains three, five, and seven nodes, respectively. To simulate different levels in the power of the association tests, for each gene with 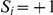, the 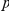 value is computed from a two-sided 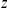 test where 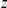 scores are randomly drawn from 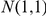, 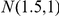 and 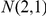, respectively, corresponding to the power 0.16 (low), 0.32 (median) and 0.51 (high) in the association tests. The 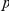 values for 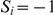 are generated randomly from Uniform(0, 1) as before.

To examine the effects of hyperparameters of the network, we consider eight priors, listed in Table 2, that roughly form four main groups indexed by numbers 1 through 4, and two subgroups indexed by letters 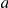 and 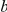. For each set of hyperparameters a Gibbs sampler is run to draw samples from the corresponding prior distribution, and we can estimate 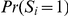, the prior mean, and 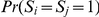 and 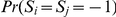 where 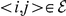, the probabilities of edge 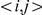 linking two nodes with identical labels. The averages of the estimated probabilities are listed in the last three columns of Table 2. The average prior means of all nodes are about 0.05, 0.15, 0.25, and 0.4, respectively for the four main groups. Roughly speaking, it means that group 1 is in favor of a small number, and group 4 a large number, while groups 2 and 3 in between, of nodes labeled with +1. Furthermore, values of 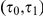 in subgroup 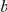 are larger than those in subgroup 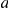, meaning that nodes with identical labels are more likely to be next to each other apriori in subgroup 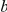 than subgroup 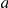, as can be seen from the last two columns in Table 2. On the other hand, because the posteriors are found to be insensitive to the hyperparameters 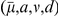 when 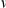 is large, they are set to (3, 1, 10, 1) as in the previous example.

**Table 2:** Eight priors.

		Hyperparameters			Estimates		
Group	Subgroup	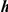	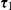	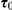	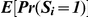	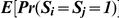	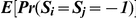
1	*a*	−3.00	0.10	0.01	0.044	0.003	0.917
	*b*	−3.00	0.25	0.10	0.049	0.043	0.923
2	*a*	−2.00	0.10	0.01	0.156	0.047	0.710
	*b*	−2.50	0.20	0.05	0.141	0.119	0.776
3	*a*	−1.25	0.05	0.01	0.250	0.081	0.563
	*b*	−3.00	0.25	0.05	0.254	0.264	0.602
4	*a*	−1.50	0.10	0.01	0.355	0.227	0.402
	*b*	−2.00	0.25	0.10	0.405	0.412	0.466

**Figure 4:**
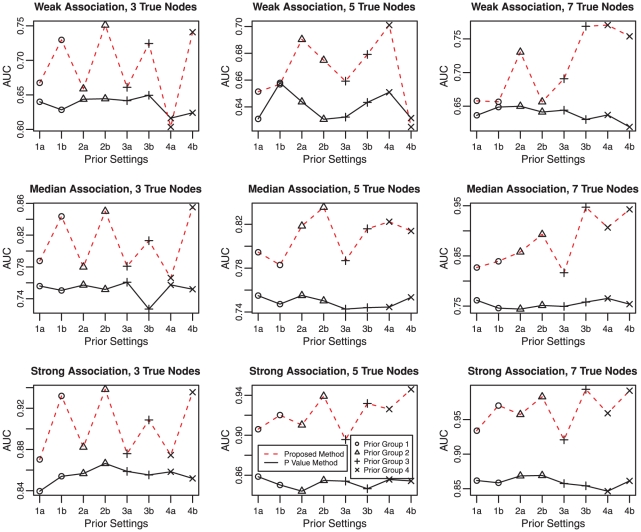
Comparison under different priors. The three rows of panels are for weak (top panel), median (middle panel), and strong (bottom panel) association signals. The three columns of panels are for three sets of truly associated genes corresponding to three (left), five (middle), and seven (right) nodes, respectively. The dotted lines link AUC of the proposed method and the solid lines connect AUC using 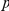 values. Circles, triangles, plus signs and crosses denote prior parameter groups 1, 2, 3, and 4, respectively.

We simulate 200 data sets for each combination of the three power settings (low, median and high) and three truly associated sets (3, 5, and 7 nodes). For each data set, we run eight Gibbs samplers using eight different hyperparameters described above. Each Gibbs sampler is run with 100 restarts and each start contains 100 steps. We compare the average AUC of 200 simulated data sets using 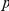 value and the posterior mean and plot the results in Figure 4. In general, the AUC of the proposed method is larger than that using 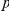 values alone. It achieves good AUC if the prior mean is close to the truth, especially when the power is low. For example, in the middle column panels where there are 5 truly associated genes, prior settings 2 and 3, favoring median number of truly associated nodes, outperform prior settings 1 and 4. Similarly, in the right panel where the true model contains 7 genes, prior settings 3 and 4, which are in favor of large models, perform better than the other prior settings. Furthermore, priors in subgroup *b* are better than subgroup *a* in general. It is not surprising because the priors in subgroup *b* encourages nodes labeled with +1 to group together, which agrees with the simulation setting.

**Figure 5:**
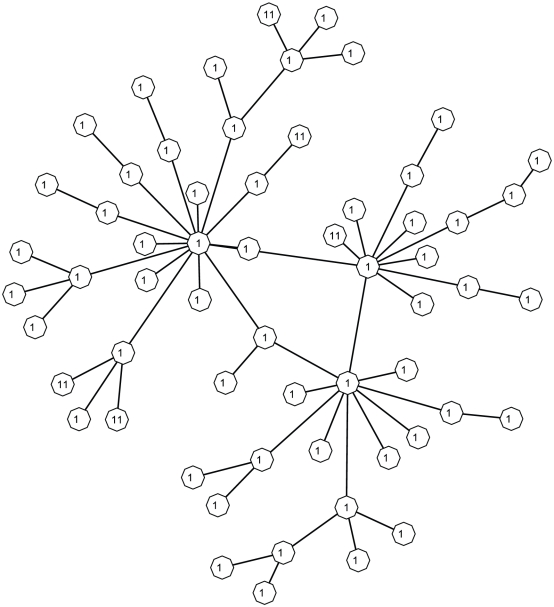
A simulated 60-node network.

To evaluate the control of the false positive rates and the false discovery rates of the proposed methods in relatively large pathways with only a few associated genes, we conduct a third simulation study based on a simulated network shown in Figure 5 that contains 60 nodes. We consider three truly associated gene sets, namely (2, 11, 19), (2, 11, 19, 41), and (2, 11, 19, 20, 41), and label them as models 1, 2 and 3 in Table 3. Similar to the previous study, we simulate 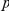 values from 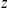 scores randomly drawn from 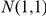, 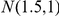 and 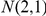, corresponding to weak, median and strong associations, respectively. Three prior settings are considered for 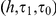, namely (−1.5, 0.15, 0.02), (−1.50, 0.10, 0.01) and (−2, 0.2, 0.01), whose average prior probability 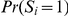 is approximately 0.2, and average prior probabilities 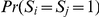 for 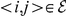 are roughly 0.13, 0.11, and 0.08, respectively. For the proposed method, we consider three decision rules. The first one (PM1) uses the posterior mean with a cut-off value 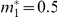 as in (5), the second one is MCP as in (4), and the third one (PM2) is the method to control the FDR at 0.1 as in (6). Then we compare them with the 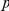 value method (P value) with a cut-off value set at 0.05 and the correction method (BH) of Benjamini & Hochberg (1995) [21]. For each scenario we simulate 100 data sets, and run a Gibbs sampler with 100 restarts where each start contains 100 iterations. For each simulated data set, we calculate the FPR, sensitivity (Sens.), FDR, and AUC as before. Table 3 lists the average values of the 100 simulation runs. In general PM1 and MCP control the FPR below the 0.05 level and have lower FDR than the 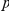 value while achieving better or similar power as the 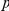 value method. In terms of controlling FDR, PM2 controls the FDR around 0.1, and it has smaller FPR or better power than the BH method in most cases when it achieves similar or better FDR.

**Table 3:** Average FPR, sensitivities, FDR, and AUC of the 60-node simulated network.

			Weak Association				Median Association				Strong Association			
Model	Method		FPR	Sens.	FDR	AUC	FPR	Sens.	FDR	AUC	FPR	Sens.	FDR	AUC
1		P Value	0.0470	0.157	0.817	0.629	0.0516	0.297	0.745	0.739	0.0523	0.533	0.621	0.863
		BH	0.0021	0.037	0.085		0.0014	0.043	0.070		0.0032	0.187	0.098	
	Prior 1	PM1	0.0412	0.167	0.790	0.657	0.0468	0.297	0.695	0.776	0.0496	0.567	0.591	0.899
		MCP	0.0370	0.150	0.768		0.0418	0.253	0.696		0.0454	0.523	0.593	
		PM2	0.0018	0.030	0.087		0.0014	0.050	0.075		0.0026	0.187	0.120	
	Prior 2	PM1	0.0404	0.163	0.792	0.653	0.0437	0.290	0.689	0.768	0.0472	0.543	0.585	0.890
		MCP	0.0375	0.150	0.779		0.0416	0.277	0.689		0.0449	0.523	0.583	
		PM2	0.0016	0.030	0.085		0.0021	0.090	0.110		0.0025	0.177	0.115	
	Prior 3	PM1	0.0300	0.143	0.688	0.690	0.0326	0.293	0.592	0.795	0.0377	0.577	0.483	0.907
		MCP	0.0253	0.140	0.648		0.0270	0.247	0.594		0.0337	0.500	0.480	
		PM2	0.0023	0.040	0.107		0.0012	0.053	0.065		0.0019	0.187	0.100	
2		P Value	0.0457	0.173	0.730	0.629	0.0514	0.330	0.668	0.738	0.0505	0.465	0.565	0.840
		BH	0.0018	0.018	0.090		0.0027	0.035	0.100		0.0038	0.178	0.094	
	Prior 1	PM1	0.0389	0.168	0.694	0.659	0.0450	0.340	0.621	0.788	0.0446	0.508	0.523	0.879
		MCP	0.0370	0.145	0.701		0.0416	0.318	0.618		0.0411	0.490	0.515	
		PM2	0.0020	0.018	0.110		0.0016	0.050	0.085		0.0016	0.178	0.075	
	Prior 2	PM1	0.0379	0.170	0.692	0.653	0.0439	0.323	0.624	0.775	0.0430	0.490	0.522	0.869
		MCP	0.0370	0.153	0.694		0.0413	0.305	0.626		0.0413	0.468	0.530	
		PM2	0.0020	0.018	0.110		0.0016	0.048	0.085		0.0020	0.158	0.095	
	Prior 3	PM1	0.0289	0.143	0.639	0.683	0.0364	0.320	0.583	0.803	0.0352	0.485	0.469	0.888
		MCP	0.0252	0.125	0.610		0.0321	0.288	0.577		0.0293	0.455	0.454	
		PM2	0.0014	0.018	0.080		0.0013	0.048	0.065		0.0027	0.205	0.108	
3		P Value	0.0478	0.148	0.756	0.648	0.0476	0.326	0.591	0.744	0.0458	0.524	0.468	0.856
		BH	0.0038	0.028	0.129		0.0015	0.050	0.051		0.0029	0.166	0.052	
	Prior 1	PM1	0.0402	0.136	0.744	0.675	0.0425	0.336	0.544	0.794	0.0409	0.584	0.422	0.897
		MCP	0.0364	0.124	0.737		0.0387	0.320	0.545		0.0373	0.568	0.403	
		PM2	0.0027	0.022	0.150		0.0011	0.056	0.053		0.0020	0.230	0.060	
	Prior 2	PM1	0.0404	0.134	0.738	0.669	0.0411	0.318	0.559	0.779	0.0398	0.556	0.427	0.886
		MCP	0.0380	0.124	0.749		0.0393	0.302	0.559		0.0375	0.546	0.414	
		PM2	0.0025	0.026	0.140		0.0009	0.052	0.045		0.0018	0.206	0.062	
	Prior 3	PM1	0.0282	0.120	0.654	0.700	0.0333	0.310	0.486	0.806	0.0315	0.556	0.381	0.904
		MCP	0.0247	0.100	0.620		0.0280	0.270	0.479		0.0276	0.540	0.358	
		PM2	0.0020	0.014	0.110		0.0011	0.046	0.055		0.0015	0.216	0.052	

### Crohn's disease data

We use one Crohn's disease [15] data set to further evaluate the performance of the proposed method. Details of this data can be found in the Materials and Methods section.

**Figure 6:**
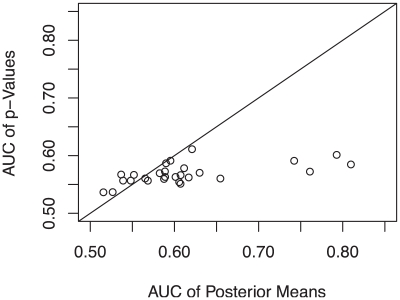
AUC comparison of rankings by 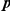 values and posterior means for Crohn's disease data. AUC values of the rankings by 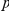 values are on the 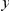 axis and that of the posterior means are on the 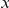 axis for pathways containing three or more target genes.

**Figure 7:**
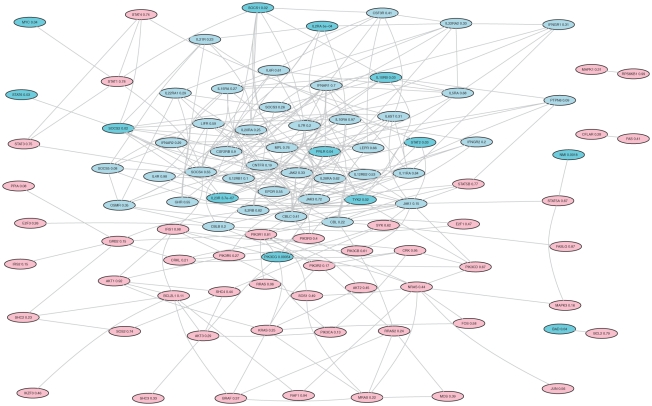
IL-2 receptor Beta chain in T cell activation pathway. Significant genes whose 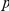 values are below 0.05 are colored in cyan, genes with improved ranks are colored in light blue and others are colored in pink.

We run our algorithm on 289 pathways that have at least 20 genes with non-missing 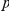 values. The hyperparameters 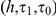 are chosen such that the average prior mean is roughly between 0.2 and 0.4 based on the simulation findings. To evaluate the performance, we consider 32 target genes that are confirmed to be related to the Crohn's disease [22]. Among these genes, 10 genes can be mapped to 66 pathways. In Figure 6 we plot the AUC values of the rankings by 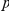 values on the 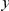 axis and posterior means on the 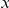 axis for pathways containing three or more target genes. A majority of AUC values are improved if genes are rank ordered by the posterior mean. The average AUC based on 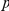 values is 0.568 while on posterior means is 0.613. To see what causes the rank changes of genes in the posterior probability, in Figure 7 we show the Human IL-2 Receptor Beta Chain in T cell Activation pathway from BioCarta. Genes in this pathway are densely connected. To aid visualization, we randomly remove some edges. Significant genes whose 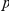 values are below 0.05 are colored in cyan, genes with improved ranks are colored in light blue and others are colored in pink. It can be clearly seen that genes colored in light blue have more connections with the significant genes, and are more heavily linked among themselves, compared to other genes in the pathway. Genes that have many interactions with each other may play important roles in the biological processes in the pathway. When they are connected to many significant genes, it might be reasonable that they are more likely to associate with the disease than other genes.

## Discussion

In this article we introduced a Bayesian method to incorporate prior knowledge of biological pathways into GWAS. This approach uses a MRF as a prior distribution to model the interactions among genes that participate in the same pathway. We showed that the posterior distribution is also a MRF and can be sampled via a Gibbs sampler. Inferences based on the posterior distribution allow us to combine data from the association study with prior information of biological pathways. In particular, this framework considers the topology of all genes in a pathway, which has not been fully utilized in many of the existing methods. The simulation studies and real data example suggest that the proposed method has higher power to identify genes associated with disease.

One limitation of the MRF model is that the Gibbs sampler tends to move around local maxima for a long time and thus can be slow in convergence to the posterior distribution. We recommend to run the Markov chain Monte Carlo with multiple random restarts, and examine the sampling distribution of network statistics, like the number of genes labeled with +1 and the proportion of edges linking genes with identical labels. In our studies, we found that a Markov chain initially moves very rapidly from its starting state, usually within the first 10 to 20 steps, before it reaches some steady states and stabilizes for a long period thereafter. We suggest running 100 Gibbs steps for each random starting state, and conducting the simulation with 100 restarts. The computing time of this scheme typically takes a few minutes on a PC for a pathway of about 30 genes. We should also mention that the characteristics of the MRF defined in (1) depend on both the hyperparameters and the structure of the network under consideration. Consequently there does not exist a set of hyperparameters that can be suitable for all pathways. To assist the specification of hyperparameters, we provide an algorithm of estimating hyperparameters based on a conditional empirical Bayes approach in Text S2. It is recommended that these values would be used in initial attempts and it would be better to test several other variants of hyperparameters, possibly through fine-tuning the initial values. It is helpful to draw samples from the prior distribution to assess the effects o f different prior settings. One limitation of pathway-based analysis is that not all the genes can be associated with pathways. It is likely with knowledge accumulation, more genes will be mapped to pathways. An R package is under construction and will be made publically available soon.

## Materials and Methods

### The MRF property of the prior distribution on pathways

The nearest neighbor Gibbs measure on gene pathways in formula (1) defines a MRF and its conditional distribution has a logistic regression form as shown below.

#### Proposition 1

The Gibbs measure in (1) is Markovian and thus defines a MRF
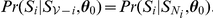



Moreover, the conditional distribution has a logistic form:
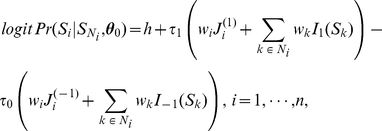
(7)


where 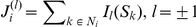. Equivalently, (7) can be rewritten as a system of linear equations:
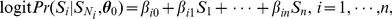
(8)where 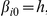


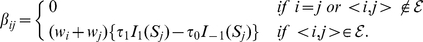



**Proof.** See Text S1.

This result shows the Markov property that the conditional distribution of 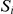, given all other node labels in the network, is equal to the conditional distribution of 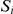 given all its neighbors. It follows immediately from (8) that if 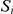 and 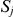 are not neighbors, then they are conditionally independent.

Now we give an interpretation of 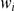. From (8), it is clear that the conditional distribution of 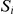 depends on the weighted sum of labels of its neighbors, with weight 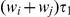 if 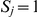 and 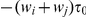 if 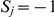. Here 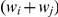 is the sum of weights on both ends of a linking edge. We set 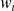 to be the square root of of 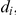 which is the degree of gene 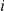. As a result, a gene that interacts with many other genes in the pathway has a large weight because it may play a central role in a biological process and thus it is likely to have a large influence.

The Markovian property of (1) can be derived directly from a more general result [18], which states that a nearest neighbor Gibbs measure is equivalent to a MRF. Our proof that is specific to (1) and is needed to derive the logistic model in (7). We note that under the setting of rectangular lattice systems, Besag [23], [24] presented a general logistic model called the auto-logistic model.

### The MRF property of the posterior distribution

To see that the posterior distribution is also a MRF, note that for node 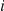,
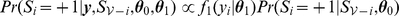


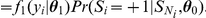



Thus, the conditional posterior distribution of 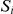 given all other nodes only depends on its neighbors, which means the posterior distribution is also a MRF. The conditional posterior log odds of 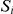 is
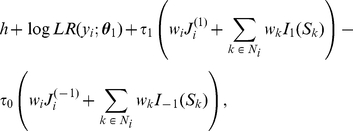
(9)


where 
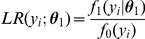
is the marginal likelihood ratio. Therefore, (9) is the product of the marginal likelihood ratio, reflecting the evidence from the data for association with the disease, and the conditional prior odds, reflecting the effect from interactions among neighboring genes from the biological pathway.

To make it clear, we can rewrite (9) in the form of a system of auto-logistic regression equations:
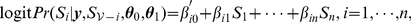
(10)where 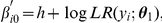


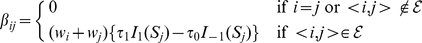



There are a few observations. First, it is easy to see that the posterior conditional logit form in (9) is the same as the prior conditional logit in (8) except its intercept is 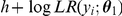. Thus, the observed log likelihood ratio provides a fixed additive effect to the prior logit. Second, the coefficient matrix is symmetric, i.e., 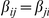. If gene 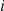 and 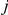 are not neighbors, then 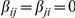 and they are conditionally independent. On the other hand, if they are neighbors, then the impact between each other is equal. Third, genes 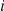 and 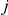 are in general correlated in their joint posterior distribution, even if they are not neighbors and are conditionally independent. Moreover, the more common neighbors they share with each other, the stronger the correlation between the two.

### The MCMC algorithm

To sample from the posterior distribution, here we implement a Gibbs sampler that is well suited for a MRF. The algorithm is described as follows. First we set an initial value for 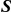, say 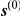. Then in step 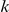, we update the labels sequentially for 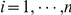 according to (10): 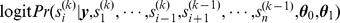


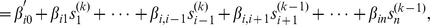
to obtain 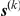 from 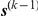. In each cycle we may want to randomize the order in which the nodes are updated.

### Crohn's disease data

The Crohn's disease [15] data set is used to evaluate the performance of the proposed method in the Results section. Crohn's disease is a type of inflammatory bowel disease characterized by chronic inflammation of discontinuous segments of the intestine. The disease is found to be related to the interaction of several factors including genetic susceptibility, the intestinal microbial flora of the patient, the patient's immune response to these microbiota, and environmental triggers [25]. It has been well established that Crohn's disease has a strong genetic component [26].

The cohort used in the analysis includes 401 cases and 433 controls. SNPs with a call rate greater than 0.9, minor allele frequency greater than 0.01, and HWE 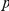 value greater than 0.001 are kept, while subjects with a call rate less than 0.95 are removed from the analysis. Finally 397 cases and 431 controls remain in the analysis. SNPs are considered being mapped to a gene if their physical locations are within 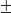
10 kb from the start or end point of the gene as given by Refseq annotation at the NCBI website. Gene level 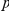 values are obtained by regressing disease status on PCA components that account for at least 85% of the variation [27]–[29]. The pathways and genes in each pathway as well as the gene-level *p* values can be found at http://bioinformatics.med.yale.edu/group/software.html.

## Supporting Information

Figure S1Estimation of 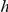 via an empirical Bayes method.(0.57 MB EPS)

Figure S2Estimation of 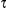 via an empirical Bayes method.(0.56 MB EPS)

Text S1 Proof of Proposition 1.
(0.12 MB PDF)

Text S2 Estimating hyperparameters through a conditional empirical Bayes approach.
(0.15 MB PDF)
