# Supplementary material for: Incorporating Biological Pathways via a Markov Random Field Model in Genome-Wide Association Studies
Source: PLoS Genet. 2011 Apr 7;7(4):e1001353. doi: 10.1371/journal.pgen.1001353 (PMC3072362; doi:10.1371/journal.pgen.1001353)
Supplement: Text S1 — Proof of Proposition 1. (0.12 MB PDF) [file pgen.1001353.s003.pdf]

Supplementary Text S1

Proof of Proposition 1

The probability function in (1) can be expressed as

$$Pr(S_1,\cdots,S_n|\boldsymbol{\theta}_0)=\frac{1}{z(\boldsymbol{\theta}_0)}\prod_{i\in\mathcal{V}}\exp(U_i),$$

where

$$U_i=hI_1(S_i)+\frac{\tau_0}{2}I_{-1}(S_i)\sum_{j\in N_i}(w_i+w_j)I_{-1}(S_j)+\frac{\tau_1}{2}I_1(S_i)\sum_{j\in N_i}(w_i+w_j)I_1(S_j).$$

The conditional probability of S_i given all other nodes in $S_{\mathcal{V}-i}$ is

$$\begin{aligned}Pr(S_i|S_{\mathcal{V}-i}) &= \frac{Pr(S_1,\cdots,S_n)}{\sum_{S_i\in\{-1,1\}}Pr(S_1,\cdots,S_i,\cdots,S_n)}\\ &= \frac{\exp(U_i)\cdot\prod_{k\in N_i}\exp(U_k)\cdot\prod_{l\in\mathcal{V}-i-N_i}\exp(U_l)}{\prod_{l\in\mathcal{V}-i-N_i}\exp(U_l)\cdot\sum_{S_i\in\{-1,1\}}\Big\{\exp(U_i)\cdot\prod_{k\in N_i}\exp(U_k)\Big\}}\\ &= \frac{\exp(U_i)\cdot\prod_{k\in N_i}\exp(U_k)}{\sum_{S_i\in\{-1,1\}}\Big\{\exp(U_i)\cdot\prod_{k\in N_i}\exp(U_k)\Big\}}. \qquad (9)\end{aligned}$$

The last step is true because for any $l\in\mathcal{V}-i-N_i$, since i and l are not neighbors, U_l is independent of S_i. Note that for any $k\in N_i$, U_k is a function of S_i:

$$\begin{aligned}U_k &= \exp\Big\{hI_1(S_k)+\frac{\tau_0}{2}I_{-1}(S_k)\sum_{j\in N_k}(w_k+w_j)I_{-1}(S_j)+\frac{\tau_1}{2}I_1(S_k)\sum_{j\in N_k}(w_k+w_j)I_1(S_j)\Big\}\\ &= \exp\Big\{hI_1(S_k)+\frac{\tau_0}{2}(w_k+w_i)I_{-1}(S_k)I_{-1}(S_i)+\frac{\tau_0}{2}I_{-1}(S_k)\sum_{j\in N_k-i}(w_k+w_j)I_{-1}(S_j)\\ &\quad+\frac{\tau_1}{2}(w_k+w_i)I_1(S_k)I_1(S_i)+\frac{\tau_1}{2}I_1(S_k)\sum_{j\in N_k-i}(w_k+w_j)I_1(S_j)\Big\}.\end{aligned}$$

Thus, (9) becomes

$$\begin{aligned}Pr(S_i|S_{\mathcal{V}-i}) &= \frac{\exp\Big\{hI_1(S_i)+\tau_0I_{-1}(S_i)\sum_{j\in N_i}(w_i+w_j)I_{-1}(S_j)+\tau_1I_1(S_i)\sum_{j\in N_i}(w_i+w_j)I_1(S_j)\Big\}}{\exp\Big\{h+\tau_1\sum_{j\in N_i}(w_i+w_j)I_1(S_j)\Big\}+\exp\Big\{\tau_0\sum_{j\in N_i}(w_i+w_j)I_{-1}(S_j)\Big\}}\\ &= Pr(S_i|S_{N_i})\end{aligned}$$

because it only depends on S_{N_i}. Thus,

$$\begin{aligned}\frac{Pr(S_i=+1|S_{N_i})}{Pr(S_i=-1|S_{N_i})} &= \frac{\exp\Big\{h+\tau_1\sum_{j\in N_i}(w_i+w_j)I_1(S_j)\Big\}}{\exp\Big\{\tau_0\sum_{j\in N_i}(w_i+w_j)I_{-1}(S_j)\Big\}}.\\ &= \exp\Big\{h+\tau_1\sum_{j\in N_i}(w_i+w_j)I_1(S_j)-\tau_0\sum_{j\in N_i}(w_i+w_j)I_{-1}(S_j)\Big\},\end{aligned}$$

which is a logistic regression model that can be rewritten as

$$\text{logit}Pr(S_i|S_{N_i}) = h + \tau_1\left(w_i J_i^{(1)} + \sum_{j\in N_i} w_j I_1(S_j)\right) - \tau_0\left(w_i J_i^{(-1)} + \sum_{j\in N_i} w_j I_{-1}(S_j)\right).$$
