# Supplementary material for: Incorporating Biological Pathways via a Markov Random Field Model in Genome-Wide Association Studies
Source: PLoS Genet. 2011 Apr 7;7(4):e1001353. doi: 10.1371/journal.pgen.1001353 (PMC3072362; doi:10.1371/journal.pgen.1001353)
Supplement: Text S2 — Estimating hyperparameters through a conditional empirical Bayes approach. (0.15 MB PDF) [file pgen.1001353.s004.pdf]

Supplementary Text S2

Estimating hyperparameters through a conditional empirical Bayes approach

The choice of the hyperparameters $\boldsymbol{\theta}_0$ and $\boldsymbol{\theta}_1$ plays an important role in the posterior inference. When prior knowledge can be obtained, for example, from past studies or theoretical developments, it should guide the selection of these hyperparameters. For example, a negative h with large magnitude will put large prior weights on $S_i = -1$. And a large τ_1 means genes with label $+1$ are very likely to be neighbors of each other. However, in the absence of such information, arbitrary choices of these hyperparameters have the risk of concentrating the prior away from the true underlying association status.

When insufficient prior knowledge is available, as is often the case in GWAS that is somewhat an initial step of screening genes, a fully Bayesian approach might be appealing that puts hyperprior distribution on these hyperparameters, to account for the intrinsic uncertainties. Then the posterior distribution can be sampled from the joint space of $(\boldsymbol{\theta}_0, \boldsymbol{\theta}_1, \boldsymbol{s})$ via MCMC. However, the potential problem of this approach is that the computation is enormous, especially when n is large, and thus only a small region of the joint sample space would be visited by the Markov chain.

To avoid the computational problem of a fully Bayesian solution, we propose an empirical Bayes method that estimates the hyperparameters from the data. More specifically, estimates of $\boldsymbol{\theta}_0$ and $\boldsymbol{\theta}_1$ can be obtained by maximizing their marginal likelihood:

$$L(\boldsymbol{\theta}_0, \boldsymbol{\theta}_1 | \boldsymbol{y}) = \sum_{\boldsymbol{s}} f(\boldsymbol{y}|\boldsymbol{s}, \boldsymbol{\theta}_1) Pr(\boldsymbol{s}|\boldsymbol{\theta}_0). \tag{10}$$

This can be seen as a fully Bayesian solution with uninformative hyper priors on the hyperparameters; but rather than averaging over all $(\boldsymbol{\theta}_0, \boldsymbol{\theta}_1)$, we choose a point estimator, namely the posterior mode, to approximate the solution. This idea is similar to the one proposed by other authors under different contexts, for example, in Bayesian variable selection [1].

However, maximizing (10) is still computationally intensive when n is large. To overcome the difficulty, one can maximize the conditional likelihood instead:

$$L^*(\boldsymbol{\theta}_0, \boldsymbol{\theta}_1, \boldsymbol{s}|\boldsymbol{y}) \propto f(\boldsymbol{y}|\boldsymbol{s}, \boldsymbol{\theta}_1) Pr(\boldsymbol{s}|\boldsymbol{\theta}_0), \tag{11}$$

which is proportional to the posterior (3). This is equivalent to maximizing the largest term, rather than the whole summation, in (10). Note that L^* is not exactly a likelihood function because $\boldsymbol{s}$ is not a parameter.

But the normalizing function $z(\boldsymbol{\theta}_0)$ in $Pr(\boldsymbol{s}|\boldsymbol{\theta}_0)$ is a function of $\boldsymbol{\theta}_0$ and is prohibitive to evaluate when n is large. So the exact solution to the maximization in (11) is infeasible. Noticing $z(\boldsymbol{\theta}_0)$ does not appear

in the conditional posterior distribution, we propose to maximize the pseudo conditional likelihood:

$$\prod_{i=1}^{n} f(y_i|s_i, \boldsymbol{\theta}_1) Pr(s_i|s_{N_i}, \boldsymbol{\theta}_0). \tag{12}$$

Certainly (12) is not a true likelihood function because each term depends on its neighboring nodes. One way of constructing a true conditional likelihood function is the coding technique [2] that chooses a subset of nodes that are not neighbors of each other so that the conditional distribution of the subset given the remaining nodes are mutually independent and can be multiplied together. Although the coding technique results in a consistent estimator, unlike the pseudo-likelihood method, it does not use all the nodes in the network. Besag [3] showed that the maximum pseudo likelihood estimator, in the setting of a general Markov random field, is consistent under mild regularity conditions. And it is more efficient than the coding estimator in simple Gaussian schemes [4].

As will be shown next, the likelihood function $f_1(y_i|\boldsymbol{\theta}_1)$ and the conditional form in (5) will greatly simplify the maximization of (12) in that they all have closed form solutions and are very easy to solve. When maximizing (12), $\boldsymbol{s}$ is treated as a missing variable. For a given $\boldsymbol{s}$, the hyperparameters $\boldsymbol{\theta}_0$ and $\boldsymbol{\theta}_1$ can be estimated separately. For $\boldsymbol{\theta}_1$, the estimate can be obtained as follows:

$$\bar{\mu} = n_1^{-1} \sum_{S_i=1} y_i,\ d = \max\big(n_1^{-1} \sum_{S_i=1} (y_i - \bar{\mu})^2, 1\big), a = \max\big(n_1^{-1} \sum_{S_i=1} (y_i - \bar{\mu})^2 / n^{-1} \sum_{i=1}^{n} (y_i - \hat{\mu})^2, 3\big), \nu = 10, \tag{13}$$

where n_1 is the number of nodes taking on the value +1, and $\hat{\mu}$ is the sample mean of n observations. Setting ν to a large value ensures the prior distribution of σ^2 has a wide spread. For $\boldsymbol{\theta}_0$, we need to maximize $\prod_{i=1}^{n} Pr(s_i|s_{N_i}, \boldsymbol{\theta}_0)$, which is equivalent to the parameter estimation of a logistic regression problem:

$$\text{logit} Pr(s_i|s_{N_i}, \boldsymbol{\theta}_0) = h + \tau_1 X_{i1} - \tau_0 X_{i0},\ \ i = 1, \cdots, n, \tag{14}$$

where the regressors are $X_{i1} = w_i J_i^{(1)} + \sum_{k \in N_i} w_k I_1(S_k)$ and $X_{i0} = w_i J_i^{(-1)} + \sum_{k \in N_i} w_k I_{-1}(S_k)$ as defined in (5). In case the MLE $\hat{\boldsymbol{\theta}}_0 = (\hat{h}, \hat{\tau}_0, \hat{\tau}_1)$ is infinite, we use a ridge estimation for logistic models [5] that subtracts a penalty term $\lambda(h^2 + \tau_0^2 + \tau_1^2)$ from the likelihood function and solves it by the Newton-Raphson algorithm. Then we can sequentially update $\boldsymbol{s}$ by maximizing (12) when fixing $(\hat{\boldsymbol{\theta}}_0, \hat{\boldsymbol{\theta}}_1)$. And these steps can be done iteratively. The algorithm is outlined as follows:

1. Set initial configuration $\boldsymbol{s}^{(0)}$;

2. In the jth iteration, for given $\boldsymbol{s}^{(j-1)}$, obtain $(\hat{\boldsymbol{\theta}}_0^{(j)}, \hat{\boldsymbol{\theta}}_1^{(j)})$ from (13) and the solution to (14);

3. Sequentially update the labels of nodes to obtain $\boldsymbol{s}^{(j)}$. For ith gene,

$$s_i^{(j)} = \arg\max_{s_i} f(y_i|s_i, \hat{\boldsymbol{\theta}}_1^{(j)}) Pr(s_i|s_{N_i}^{(j-1)}, \hat{\boldsymbol{\theta}}_0^{(j)}) \prod_{k \in s_{N_i}} Pr(s_k^{(j-1)}|s_i, s_{N_k - i}^{(j-1)}, \hat{\boldsymbol{\theta}}_0^{(j)}),$$

which is trivial to solve because $f(\cdot)$ is a normal density, and the conditional distributions have logistic forms. Besides, only node i and its neighbors $k \in s_{N_i}$ need to be evaluated. It is easy to see that the above optimization is equivalent to comparing the values of (12) evaluated at $s_i = +1$ and $s_i = -1$ while holding all other nodes at $s_k^{(j-1)}$, $k \neq i$, and fixing the hyperparameters at $(\hat{\boldsymbol{\theta}}_0^{(j)}, \hat{\boldsymbol{\theta}}_1^{(j)})$;

4. Repeat steps 2 and 3.

It is clear that in each step of every iteration, the objective function (12) is non-decreasing. So the algorithm stops when the pseudo-likelihood does not increase or reach a pre-specified iteration number. Similar to the ICM, this algorithms seems to run very rapidly, but it tends to reach a local maximum that is not globally optimal. Thus, multiple restarts with various initial configurations are recommended.

Simulation study

We conduct a simulation study to test the performance of the above algorithm. The network we use is shown in Figure 3. The prior parameters are set at h=(-0.1, -0.2, -0.3, -0.4) and $\tau_0 = \tau_1 = \tau$=(0.1, 0.2, 0.3, 0.4). Each of the 16 combinations of h and τ determines a prior distribution from which a configuration of node labels are randomly drawn. For a given configuration of labels, p values are simulated by first drawing random values, from $N(0,1)$ for $S_i = -1$ and $N(1,1)$ for $S_i = 1$, that are subsequently converted to p values assuming they are from $N(0,1)$. Finally the hyperparameters h and τ are estimated by the above algorithm. The ridge parameter is set at $\lambda = 0.5$. This process is repeated for 4,000 times, and the mean and standard error of $\hat{h}$ and $\hat{\tau}$ are plotted in Figure S1 and S2, respectively. The estimator $\hat{h}$ seems to be unbiased when $h = -0.4$ and -0.3, and it is biased low at -0.2 and -0.1. And $\hat{\tau}$ has small bias when $\tau = 0.1$ and 0.2 but is noticeably biased low for τ=0.3 and 0.4. The bias is likely caused by the penalty on the norm of hyperparameters in the ridge logistic regression.

References

1. George EI, Foster DP (2000) Calibration and empirical Bayes variable selection. Biometrika 87: 731–747.

2. Besag J (1974) Spatial interaction and the statistical analysis of lattice systems. Journal of the Royal Statistical Society Series B (Methodological) 36: 192-236.

3. Besag J (1975) Statistical analysis of non-lattice data. The Statistician 24: 179–195.

4. Besag J (1977) Efficiency of pseudolikelihood estimation for simple Gaussian fields. Biometrika 64: 616-618.

5. Cessie SL, Houwelingen JCV (1992) Ridge estimators in logistic regression. Applied Statistics 41: 191–201.
